# One Actor, Multiple Roles: The Performances of Cryptochrome in *Drosophila*

**DOI:** 10.3389/fphys.2020.00099

**Published:** 2020-03-05

**Authors:** Milena Damulewicz, Gabriella M. Mazzotta

**Affiliations:** ^1^Department of Cell Biology and Imaging, Jagiellonian University, Kraków, Poland; ^2^Department of Biology, University of Padua, Padua, Italy

**Keywords:** cryptochrome, *Drosophila*, circadian clock, phototransduction, circadian plasticity, light-independent activity

## Abstract

Cryptochromes (CRYs) are flavoproteins that are sensitive to blue light, first identified in *Arabidopsis* and then in *Drosophila* and mice. They are evolutionarily conserved and play fundamental roles in the circadian clock of living organisms, enabling them to adapt to the daily 24-h cycles. The role of CRYs in circadian clocks differs among different species: in plants, they have a blue light-sensing activity whereas in mammals they act as light-independent transcriptional repressors within the circadian clock. These two different functions are accomplished by two principal types of CRYs, the light-sensitive plant/insect type 1 CRY and the mammalian type 2 CRY acting as a negative autoregulator in the molecular circadian clockwork. *Drosophila melanogaster* possesses just one CRY, belonging to type 1 CRYs. Nevertheless, this single CRY appears to have different functions, specific to different organs, tissues, and even subset of cells in which it is expressed. In this review, we will dissect the multiple roles of this single CRY in *Drosophila*, focusing on the regulatory mechanisms that make its pleiotropy possible.

## Introduction

Cryptochromes are highly conserved proteins belonging to the flavoprotein superfamily, identified in species from all three domains of life (Chaves et al., 2011). They are structurally related to photolyases (Müller and Carell, 2009), evolutionarily conserved flavoproteins that catalyze light-dependent DNA repair (Todo, 1999; Sancar, 2003). Cryptochromes and photolyases bind the same cofactors: the flavin adenine dinucleotide (FAD) and a secondary cofactor such as methenyltetrahydrofolate (MTHF), deazariboflavin, or others (Sancar, 2003). Cryptochromes have essentially lost their DNA repair activity and have acquired a very divergent C-terminal domain, intrinsically unstructured (Hemsley et al., 2007) and critical for light signaling (Chaves et al., 2011). A class of cryptochromes, CRY-DASH (*Drosophila*, *Arabidopsis*, *Synechocystis*, and *Homo*), with structural and photochemical properties more similar to photolyases and residual single-stranded DNA repair activity, has been described in bacteria, plants, and animals (Selby and Sancar, 2006; Pokorny et al., 2008).

Cryptochromes are involved in the regulation of circadian clocks, but they also display several signaling functions, ranging from growth and development in plants (Yang et al., 2017) to putative magnetoreception in animals (Ritz et al., 2000). From a circadian perspective, animal cryptochromes can be essentially divided into two classes of proteins: light-responsive type 1 (from invertebrates), involved in clock entrainment, and light-insensitive type 2 (mainly found in vertebrates but also in some insects), acting as transcriptional repressors in the central clock mechanism (Chaves et al., 2011). In recent years, new types of CRY/PHR (cryptochromes/photolyases) have also been described, providing evidences for the large functional diversity of this group of proteins (for a comprehensive description and phylogenetic classification, refer to Ozturk, 2017).

## Structure and Photoactivation

**FIGURE 1:**
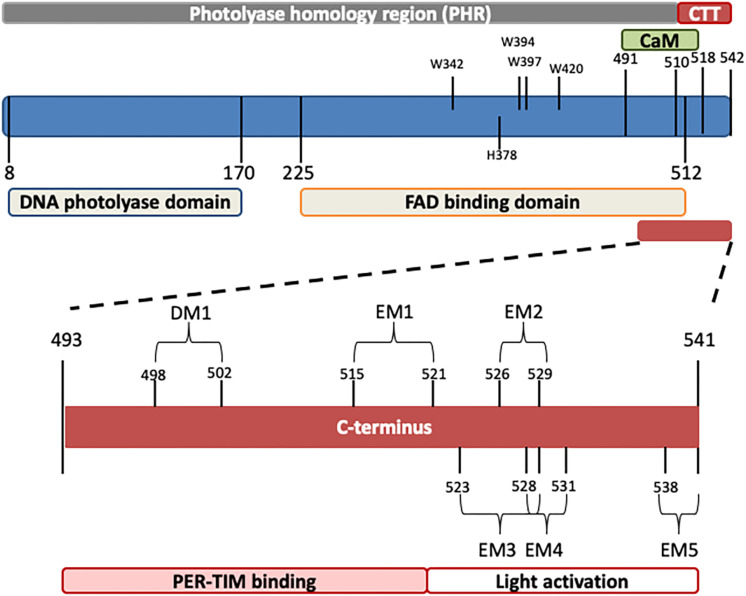
Schematic representation of *Drosophila* CRY. The photolyase-like and FAD binding domains (below) as well as the calmodulin binding motif (CaM) and the C-terminus tail (CTT) (above) are indicated. In the C-terminus, relevant domains are also depicted. Numbers indicate position (amino acids). For details, see Table 1.

*Drosophila* CRY, defined as type 1 cryptochrome (Yuan et al., 2007; Öztürk et al., 2008), is a photoactive pigment whose action spectrum peaks in the UV-A range (350–400 nm) with a plateau in the near blue (430–450 nm) (VanVickle-Chavez and Van Gelder, 2007). The 542-amino-acid (aa) protein harbors two different domains (Table 1): an N-terminal photolyase homology region (PHR) and a C-terminus tail (CTT), unique in its sequence, responsible for mediating phototransduction (Busza et al., 2004; Dissel et al., 2004; Hemsley et al., 2007; Figure 1). The CTT forms a helix structure that binds alongside the main body of the PHR domain establishing contacts with the FAD binding pocket, mimicking the damaged DNA photolyase–DNA interaction (Zoltowski et al., 2011; Czarna et al., 2013; Levy et al., 2013; Masiero et al., 2014; Lin et al., 2018). Upon illumination with blue light (440 nm), the CRY FAD cofactor is reduced to the anionic semiquinone (ASQ) state by a fast electron transfer involving four conserved tryptophan residues (W420, W397, W342, and W394). FAD photoreduction induces conformational changes in the Trp tetrad, which result in the displacement of the CTT from the PHR domain and consequent protein activation (Zoltowski et al., 2011; Czarna et al., 2013; Levy et al., 2013; Vaidya et al., 2013; Masiero et al., 2014; Lin et al., 2018). However, the Trp-tetrad-dependent photoreduction and circadian photic resetting were suggested to be independent of each other (Ozturk et al., 2014).

**TABLE 1:** Functional domains and relevant residues in the CRY protein.

	**Position (amino acids)**	**Motifs**	**Function**
N-terminus	1–492		
DNA photolyase domains	8–170		Light detection
FAD binding domain	225–512		
TRP tetrad	W342, W394, W397, W420		Fast electron transfer Conformational change
H378	H378		Stabilization of CTT in the resting state conformation in the dark
C-terminus	493–541		
DM1	498–502	Interaction motif	
CaM binding motif	491–518		Ca^2+^-dependent Calmodulin binding
C-terminus tail (CTT)	510–542		
EM1	515–521	Proline-directed kinase phosphorylation site	PER and TIM binding
EM2	526–529	TRAF2 ligand motif and part of a putative phosphorylation site	Light-dependent activation
EM3	523–529	Casein kinase 2 and cAMP-dependent protein kinase A (PKA) phosphorylation site	Light-dependent activation Phosphorylation of S526 induces the modulator replacement by TIM/PER
EM4	528–531	PDZ binding motif	Light-dependent activation E530-repressor binding
EM5	538–541	PDZ binding motif	Light-dependent activation

Very recently, a role for the Trp triad (W420, W397, and W342) in circadian photoentrainment of locomotor activity rhythm was tested *in vivo*, by analyzing the behavioral response to moderately and very low light. While W420Y and W397Y CRY flies were predominately arrhythmic (similar to wild type), transgenic flies expressing W342Y CRY showed high levels of rhythmicity and long periods, similar to *cry*^0^ flies (Dolezelova et al., 2007; Baik et al., 2019).

Molecular dynamics (MD) simulations have suggested that the CTT detachment is also a result of changes in the hydrogen bonding network due to protonation of a conserved His residue (His378), located between the CTT and the flavin cofactor (Ganguly et al., 2016). H378 stabilizes the CTT in the resting-state conformation in the dark; light induces a series of conformational changes from nanoseconds to milliseconds that lead to the formation of the final signaling state, which depends on pH and requires uptake of a proton (Berntsson et al., 2019). MD simulations have also suggested for the FAD cofactor roles other than photoreduction and CRY activation: the FAD presence would confer to the receptor a more fluctuation-prone behavior, thus decreasing the amount of necessary light input energy for CRY activation (Masiero et al., 2014). Recent studies performed on a longer timescale have revealed that following photoactivation, FAD is released from the FAD-binding pocket, providing evidence that CRY undergoes an inactivation reaction rather than a photocycle (Kutta et al., 2018), in agreement with the reported irreversible nature of the light-induced conformational changes (Ozturk et al., 2009; Kattnig et al., 2018; Lin et al., 2018). The active form of CRY is then able to bind the circadian components TIMELESS (Ceriani et al., 1999) and PERIOD (Rosato et al., 2001).

The CTT of CRY has been extensively studied, and a combination of *in silico* analyses and experimental validation has revealed the presence of an intrinsically disordered region containing several interaction motifs that turn this tail into a hot spot for molecular interactions (Hemsley et al., 2007; Mazzotta et al., 2013; Masiero et al., 2014). It can be divided into two subregions: one (493–520 aa) required for the interaction with PER and TIM (Hemsley et al., 2007) and the other (521–542 aa) specifically involved in the light activation of the CRY protein (Rosato et al., 2001; Busza et al., 2004; Dissel et al., 2004). The absence of part of the CTT (aa 521–540_CRYΔ or aa 524–542_CRY^M^) results in constitutive activation of the protein (Rosato et al., 2001; Busza et al., 2004). In this state, CRY may bind TIM and PER in the absence of light (Rosato et al., 2001); in flies overexpressing CRYΔ in the pacemaker neurons, the accumulation of clock proteins is reduced, and their subcellular distribution altered. At a behavioral level, these flies display long periods of locomotor activity rhythms in constant darkness (Dissel et al., 2004). This is reminiscent of the similarly long period shown by wild-type flies exposed to constant light of low intensity (Konopka et al., 1989; Dissel et al., 2004) (see Table 2). The first subregion of CRY CTT (aa 515–521) harbors the interaction motifs DM1 (DILIMOT database, Neduva and Russell, 2005) and EM1 (ELM database (Gould et al., 2009) and contains a proline-directed kinase phosphorylation site (Hemsley et al., 2007). In the second subregion, four putative ELM interaction motifs have been identified (EM2–EM5) (Hemsley et al., 2007). EM2 (526–529) is a TRAF2 ligand motif and part of a putative phosphorylation site, EM3 (523–529) contains putative phosphorylation sites for casein kinase 2 (CK2) and cAMP-dependent protein kinase A (PKA), EM4 (528–531) and EM5 (538–541) are PDZ binding motifs (Hemsley et al., 2007).

**TABLE 2:** *Cry* mutants.

**Mutant**	**Defect**	**Molecular**	**Behavioral**	**References**
*cry*^b^	Missense mutation (D412N) in the conserved FAD binding domain	No cycling of mRNA; very low protein levels No cycling of *per*/*tim* in peripheral clocks Light-independent interaction with TIM No light-dependent degradation	No phase shift in response to light pulses Free-running circadian rhythms in constant light	Stanewsky et al., 1998; Emery et al., 2000; Krishnan et al., 2001; Levine et al., 2002; Busza et al., 2004; Yoshii et al., 2004; Rieger et al., 2006
*cry*^M^	Deletion of C-terminus (amino acids 524–542)	Low protein levels No light-dependent degradation. Light-independent interaction with TIM	Free-running circadian rhythms in constant light	Busza et al., 2004
*cry*^0^	Knockout	Reduced *per* oscillation in wings and antennae under LD conditions	Two separate circadian components in constant light	Dolezelova et al., 2007
*cryΔ*	Deletion in C-terminus (amino acids 521–540)	Low protein levels No light-dependent degradation Reduced PER/TIM levels, cycling amplitude, and phosphorylation status Impaired nuclear localization of TIM/PER in LN_v_s Light-independent interaction with PER/TIM	Longer free-running period Entrainment defects	Rosato et al., 2001; Dissel et al., 2004
*cry*^out^	Deletion of N-terminal (amino acids 1–96)		Free running in LD Entrainment to temperature cycles	Yoshii et al., 2008, 2010

**FIGURE 2:**
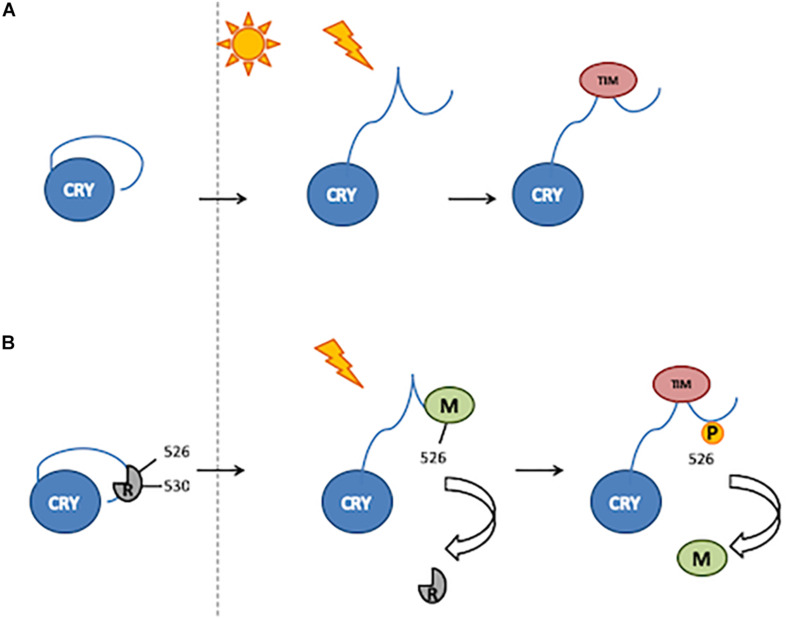
Two mechanisms of CRY activation. **(A)** Light induces a conformational change resulting in the release of CTT, thus enabling TIM binding. **(B)** In the darkness, a putative repressor (R) binds to the 530 residue and blocks the 526 position. After light exposure, the repressor is released and a modulator (M) binds to the 526 position. Phosphorylation of the 526 residue is involved in modulator release and thus TIM binding.

An alternative model proposed for the light activation of CRY involves the binding of CTT by still unknown factor(s), acting as repressor(s) in the dark and released upon light exposure (Rosato et al., 2001; Hemsley et al., 2007). Residue Glu530 (E530) might be involved in the binding of a repressor in the darkness, which would block the Ser526 (S526) residue in the TRAF2 ligand motif, thus inhibiting further bindings. After light exposure, the repressor would be released, and modulator proteins might bind to TRAF2 (Hemsley et al., 2007; Figure 2).

## CRY and Circadian Clock Resetting

**FIGURE 3:**
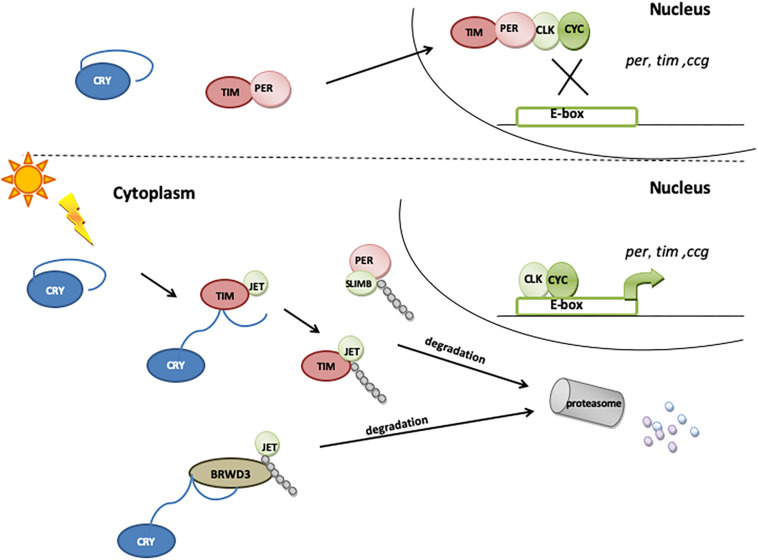
The role of CRY in molecular clock resetting. In the presence of light, CRY binds TIM and promotes its degradation via proteasome by a mechanism that involves the F-box protein JETLAG (JET). When exposed to light, CRY also becomes a substrate for JET and for Ramshackle (BRWD3), which initiates its ubiquitination and degradation in proteasome.

*Drosophila* CRY acts as photoreceptor involved in the light synchronization of the molecular circadian clock machinery (Stanewsky et al., 1998; Helfrich-Förster et al., 2001) based, as in virtually all eukaryotes, on interlocked feedback loops. In the *Drosophila* main negative feedback loop, PERIOD (PER) and TIMELESS (TIM) proteins act as negative elements, inhibiting the transcription of their own genes. Their expression is activated by CLOCK (CLK) and CYCLE (CYC): in the evening, they dimerize, enter the nucleus and bind to the E-box, thus inducing the expression of *per*, *tim*, and other clock-controlled genes (*ccg*). PER and TIM proteins accumulate in the cytoplasm, and late at night, they dimerize and translocate to the nucleus, where they bind to CLK/CYC and inhibit their activity, repressing the transcription of *ccg* [for a review, refer to Özkaya and Rosato (2012) and Figure 3]. The second feedback loop is based on rhythmic *vrille* (*vri*) and *Pdp1*ε (PAR-domain protein 1) expression (McDonald and Rosbash, 2001; Ueda et al., 2002). Both genes are transcribed with the same phase, but while VRI protein expression quickly follows that of its mRNA, PDP1ε starts to accumulate 3–6 h later (Cyran et al., 2003). VRI forms homodimers that bind to the V/P box located in the promoters of morning genes (i.e. *clk* and *cry*), blocking their transcription (Cyran et al., 2003; Glossop et al., 2003). After 3–6 h, PDP1ε starts to compete with VRI for the V/P box binding position, and because of a higher affinity, it releases the inhibitor and activates the expression of controlled genes in the late night (Cyran et al., 2003). This mechanism ensures the circadian expression of CRY, with mRNA peaking at the end of the day and maximum levels of protein level during the night (Emery et al., 1998). This rhythm of RNA expression is maintained in constant darkness conditions (DD), although with decreased amplitude, while CRY protein levels in DD increase continuously during the subjective day and night (Emery et al., 1998). In constant-light conditions, CRY is overactivated, which causes the amplitude of TIM and PER cycling to be reduced and TIM phosphorylation status to be attenuated (Marrus et al., 1996). As a consequence, flies are arrhythmic or exhibit longer period of locomotor activity rhythm, depending on the intensity of light (Konopka et al., 1989).

The *Drosophila* pacemaker operates within a circuitry consisting of a network of 150 clock neurons divided into nine groups: four groups of dorsal neurons (DN1_a_, DN1_p_, DN2, and DN3) and five groups of lateral neurons, further divided into lateral posterior neurons (LPNs), ventral lateral neurons (LN_v_s), and dorsal lateral neurons (LN_d_s). The ventral lateral neurons are classified, based on their relative size, into small and large (s-LN_v_s and l-LN_v_s, respectively), and fifth s-LN_v_ (Miyasako et al., 2007; Hermann-Luibl and Helfrich-Förster, 2015). s-LN_v_ and l-LN_v_ express a pigment-dispersing factor (PDF), a neuropeptide involved in intercellular communication between clock neurons (Shafer et al., 2008; Yoshii et al., 2009).

Cryptochrome is expressed in a subset of clock neurons (all four s-LN_v_s, all four l-LN_v_s, the fifth s-LN_v_, three of the six LN_d_s, and some of the DN1s), enabling them to directly perceive photic information (Shafer et al., 2006; Benito et al., 2008; Yoshii et al., 2008; Damulewicz and Pyza, 2011; Fogle et al., 2011). Upon light exposure, CRY binds to TIM, promoting its degradation (Ceriani et al., 1999; Koh et al., 2006b; Peschel et al., 2009). As the presence and binding of TIM are essential for PER stability, the light-induced degradation of TIM releases the PER–TIM mediated transcriptional repression, hence synchronizing the circadian clock to light–dark cycles (Ishida et al., 1999; Helfrich-Förster, 2005). CRY is also rapidly degraded in the presence of light through the proteasome (Lin et al., 2001; Sathyanarayanan et al., 2008): the light-dependent CRY–TIM complex is bound by JETLAG (JET), which promotes TIM ubiquitination and degradation. In the absence of TIM, CRY binds JET (Peschel et al., 2009) or Ramshackle (BRWD3) (Ozturk et al., 2013) or both. JET is a component of a Skp1-Cullin/F-Box (SCF) E3 ubiquitin ligase complex and functions as a substrate receptor for CRL1 E3 ligase (Koh et al., 2006b), while BRWD3 is a substrate receptor for CRL4 E3 ligase (Ozturk et al., 2013). JET and BRWD3 initiate CRY ubiquitination and degradation in the proteasome (Figure 3). The light dependence of this binding, which is enhanced in the absence of TIM, leads to a rapid decrease in CRY levels during the day, just after TIM degradation. This way, CRY resets the molecular clock and entrains the oscillator to light conditions.

Besides its relevance for circadian photo-synchronization, the CRY–TIM interaction has also important functional implications in the clock adaptation to seasonal environments. Indeed, natural variants of TIM known to trigger seasonal responses as a function of photoperiod show, at a molecular level, differential affinity for CRY (Boothroyd et al., 2007; Sandrelli et al., 2007; Tauber et al., 2007; Montelli et al., 2015).

Interestingly, CRY interacts also with PER, detecting PER as a possible pacemaker target of the cryptochrome: in a yeast two-hybrid assay, this interaction is light dependent, while in S2 cells, the physical association between CRY and PER is independent of light (Rosato et al., 2001).

From the first described CRY mutant, *cry*^b^, a missense mutation in the FAD binding site (Stanewsky et al., 1998), several *cry* mutations have been shown to affect circadian light response (for a detailed description, refer to Table 2). Conversely, CRY overexpression increases flies’ sensitivity to low-intensity light (Emery et al., 1998).

The *Drosophila* circadian clock can be readily synchronized by temperature cycles with an amplitude of 2–3°C (Glaser and Stanewsky, 2005; Yoshii et al., 2005; Goda et al., 2014; Currie et al., 2009), and different subsets of clock neurons are specifically involved in mediating clock synchronization at high or low temperatures (Zhang Y. et al., 2010; Gentile et al., 2013). Interestingly, among the various subsets of clock neurons, those more easily synchronized by temperature are the ones that do not express CRY (Yoshii et al., 2010; Gentile et al., 2013; Yadlapalli et al., 2018), and consistent with this finding, removal of CRY from clock neurons increases flies’ ability to synchronize to temperature cycles (Gentile et al., 2013). Thus, in clock neurons, CRY plays an important role in counteracting the effects of temperature cycles on the molecular circadian clock, thus contributing to the integration of different zeitgebers.

## CRY and Circadian Pacemaking

Cryptochrome also acts as a circadian transcriptional repressor necessary for the daily cycling of peripheral circadian clocks. Indeed, the endogenous rhythms of olfactory responses are severely reduced or abolished in *cry*^b^ mutants, as well as molecular oscillations of *per* and *tim* during and after entrainment to light–dark cycles (Krishnan et al., 2001). The same *cry*^b^ mutation dramatically affects the pattern of PER and TIM oscillation in Malpighian tubules (MTs), where both proteins display very low levels during most of the DD cycle (Ivanchenko et al., 2001). By contrast, the same mutation does not affect circadian oscillator functions in central circadian pacemaker neurons (Ivanchenko et al., 2001; Stanewsky et al., 1998). Moreover, the expression level of genes activated by CLK/CYC is reduced in *cry*^b^ mutant eyes (Collins et al., 2006; Damulewicz and Pyza, 2011); on the other hand, CRY and PER co-expression in the compound eyes represses CLK/CYC activity (Collins et al., 2006). This role of CRY as a clock component seems limited to peripheral oscillators.

Besides this role as a circadian repressor, an involvement of CRY in the posttranscriptional control of the circadian clock can also be hypothesized. Indeed, we have recently shown that CRY interacts with BELLE (Cusumano et al., 2019), a DEAD-box RNA helicase essential for viability and fertility (Johnstone et al., 2005), and plays important functions in RNA metabolism, from splicing and translational regulation to miRNA and siRNA pathways (Worringer et al., 2009; Pek and Kai, 2011; Ihry et al., 2012). We have observed an involvement of BELLE in circadian rhythmicity and in the piRNA-mediated regulation of transposable elements, suggesting that this specific posttranscriptional mechanism could be in place to ensure proper rhythmicity (Cusumano et al., 2019).

## CRY and Magnetoreception

In several organisms, circadian rhythms are influenced by little changes in the intensity of the Earth’s magnetic field. In particular, a low-frequency electromagnetic field shows a pronounced 24-h oscillation (König, 1959), and therefore, it could act as a geophysical synchronizer for the circadian clock (Yoshii et al., 2009). Insects detect the geomagnetic field using photochemical reactions: photon absorption by pigment molecules induces the transfer of an electron from a donor to an acceptor molecule, generating a donor–acceptor couple, each molecule containing one unpaired electron, called radical pair in singlet state (antiparallel spin orientation). The two unpaired electrons are at a proper distance to undergo transition to the triplet state (parallel orientation), and the geomagnetic field can influence the interconversion between single and triplet states of the radical pair (Ritz et al., 2000).

In *Drosophila*, CRY is a good candidate for sensing small changes in the magnetic field. In fact, in CRY, radical pairs are formed between the FAD cofactor and proximate tryptophan and/or tyrosine residues within a conserved Trp triad (W342, W397, and W420) (Zoltowski et al., 2011; Czarna et al., 2013; Levy et al., 2013). The photon is absorbed by the pigment molecule, and then one electron is transferred from the triad following electron excitation of the FAD and consequent protonation and deprotonation (Dodson et al., 2013). Radical formation activates CRY, which changes its conformation. A reverse reaction (reoxidation) restores the fully oxidized (inactive) form of CRY in the dark. This process can also generate magnetically sensitive radical pairs (superoxide and peroxide radicals and flavin radicals) (Dodson et al., 2013). There is also evidence that CRY is co-expressed and, in the presence of light and the magnetic field, forms a stable complex with CG8198 [Lethal (1) G0136], thereafter named MagR (Qin et al., 2016), an iron–sulfur cluster assembly protein involved in iron metabolism and required for proper circadian rhythmicity (Mandilaras and Missirlis, 2012).

*Drosophila* behavior is influenced by the magnetic field. Indeed, in a binary-choice behavioral assay for magneto-sensitivity, flies exhibit a naïve avoidance of the magnetic field under full-spectrum light but did not respond when UV-A/blue light (<420 nm) was blocked (Gegear et al., 2008). This response was also lost in *cry* mutants, clearly indicating that CRY is directly involved in the light-dependent magnetic sensing in *Drosophila* (Gegear et al., 2008). The electromagnetic field influences the period length of the locomotor activity, as a result of enhanced CRY signaling. Indeed, electromagnetic field application caused lengthening of the circadian period of locomotor activity, and this effect was greater when the flies were exposed to constant blue light, reasonably as a result of an enhanced CRY function upon blue-light activation (Yoshii et al., 2009). Furthermore, *cry* mutants showed no magnetic field sensitivity for period changes, whereas flies overexpressing CRY were magnetically oversensitive (Ritz et al., 2010). Further analyses of low-frequency electromagnetic field-induced changes on circadian period and activity levels have shown that the terminal tryptophan of the Trp triad (W342) is not necessary for field responses, but a mechanism different from radical pairs involving the Trp triad might be used by CRY to sense the electromagnetic field (Fedele et al., 2014a). Indeed, superoxide radicals and ascorbic acid could form a radical pair with the FAD (Müller and Ahmad, 2011; Lee et al., 2014). However, deletion of the CRY C-terminus weakens the period changes in response to the magnetic field, while the N-terminus increases hyperactivity (Fedele et al., 2014a).

Climbing activity (negative geotaxis) is disrupted by a static electromagnetic field (Fedele et al., 2014b). This effect is observed after blue-light exposure but is not present in red light, indicating the involvement of light-activated CRY (Fedele et al., 2014b). Mutation of the terminal Trp in the triad (W342) in CRY does not affect magnetoreception (Gegear et al., 2010; Fedele et al., 2014b), but C-terminus deletion disrupts the fly’s response to the electromagnetic field (Fedele et al., 2014b). *cry* mutants show decreased climbing ability, which can be rescued by overexpressing CRY in LN_d_ clock neurons (Rakshit and Giebultowicz, 2013), antennae, and eyes (R8 photoreceptors of pale ommatidia, R8 yellow ommatidia, H-B eyelet, or R7 cell) (Fedele et al., 2014b).

Cryptochrome is involved also in the modulation of other responses of *Drosophila* to the magnetic field. The courtship activity of wild-type males is significantly increased when they were exposed to a ≥20-Gauss static magnetic field (Wu et al., 2016), but not in *cry*-deficient mutants (*cry*^b^ and *cry*^M^) and in flies with CRY RNAi-mediated knockdown in *cry*-expressing neurons (Wu et al., 2016). Nevertheless, the phenotype is rescued when CRY is genetically expressed under the control of *cry*-Gal4 (Wu et al., 2016).

The magnetic field influences also the seizure response in *Drosophila*, specifically the recovery time of larvae from an electric shock (Marley et al., 2014). Indeed, a stronger seizure phenotype is observed after blue light or magnetic field exposition, and the lack of this effect in either *cry*^0^ mutants or in orange light (590 nm) clearly indicates it to be CRY dependent (Marley et al., 2014). Moreover, this strong seizure phenotype is associated with increased synaptic excitation in the locomotor circuitry, as it may be blocked by antiepileptic drugs (Marley and Baines, 2011; Lin et al., 2012). Indeed, the CRY- and light-dependent magnetic field modulates the action potential firing of individual neurons, by increasing input resistance and depolarization of the membrane potential of “anterior Corner Cell” (aCC) and “Raw Prawn 2” (RP2) motoneurons (Giachello et al., 2016).

The ability of cryptochromes to form radical pairs upon photoexcitation makes them excellent candidate proteins for light-dependent magnetoreception also in other organisms.

The vertebrate-like Cry2 is involved in the response to magnetic field of two species of cockroaches, the American cockroach, *Periplaneta americana* (which most likely contains only Cry2), and *Blattella germanica*, which has both CRY types (Bazalova et al., 2016). Cry2 is expressed in laminal glia cells underneath the retina and is necessary for sensing the directional component of the magnetic field (Bazalova et al., 2016).

The night-migratory European robins (*Erithacus rubecula*) possess four different cryptochromes, but only Cry4 is predicted to be the magnetoreceptive protein (Günther et al., 2018). Cry4 is expressed in every cell type within the retina, at significantly higher levels during the migratory season compared to the non-migratory season. Moreover, the modeled structure revealed a high similarity with *Drosophila* CRY, also in the position of residues important for FAD binding (Kutta et al., 2017; Günther et al., 2018).

## CRY in the Visual System

In addition to the pacemaker neurons, CRY is also present in non-clock cells in the anterior brain, in the glia cells located between the central brain and the optic lobe, as well as in the terminals of photoreceptor cells R7 and R8 (Yoshii et al., 2008; Damulewicz and Pyza, 2011). In photoreceptor cells, it is mainly involved in the functioning and localization of the phototransduction cascade proteins (Mazzotta et al., 2013; Schlichting et al., 2018). The visual cascade proteins are located in the rhabdomeres, densely packed microvilli formed by evaginations of the photoreceptors’ plasma membrane. These are arranged in a multiprotein complex called Signalplex, organized by the inactivation-no-afterpotential D (INAD), a PDZ [postsynaptic density protein (PSD95), *Drosophila* disc large tumor suppressor (Dlg1), and zonula occludens-1 protein (zo-1)] domains-containing protein [reviewed by Hardie and Juusola (2015)].

In the photoreceptor cells, CRY binds to INAD, which, in turn, enables the interaction with other phototransduction components (Mazzotta et al., 2013). INAD binds the neither-inactivation-nor-afterpotential C (NINAC) myosin III, involved in the shuttling of signaling proteins (Gqα and arrestin 2) from the cell bodies to the rhabdomeres [reviewed by Montell (2012)] and in the inactivation of metarhodopsin by speeding up the binding of arrestin (Liu et al., 2008). INAD/NINAC interaction allows binding of the complex to F-actin filaments (Montell, 2007), which contributes to maintaining the rhabdomere structure (Arikawa et al., 1990; Porter et al., 1992). Especially in the dark, INAD binds to TRP channels and keeps them in the rhabdomeres, ready for activation, while after light adaptation, TRP channels translocate into the cell body (Montell, 2007).

**FIGURE 4:**
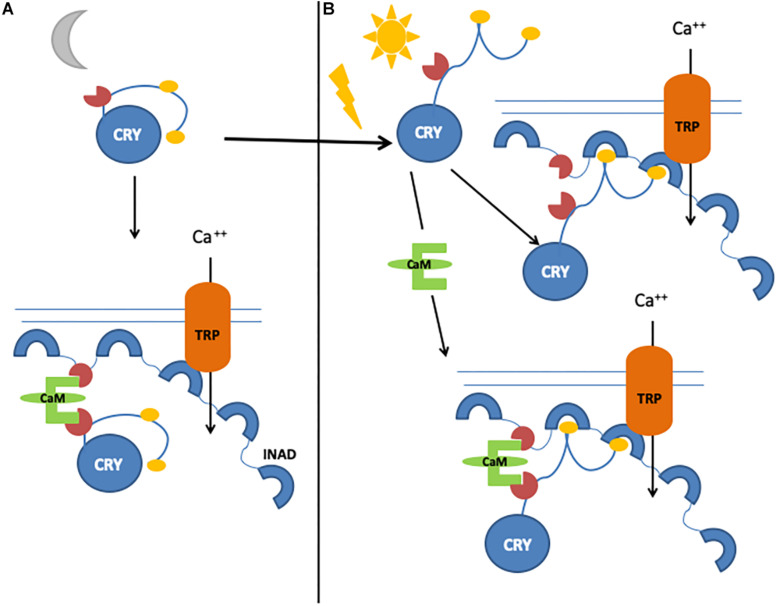
Graphical representation of CRY/calmodulin interaction mechanisms. **(A)** Light-independent, CaM-dependent pathway. Inactive CRY binds to CaM, which allows the formation of a ternary complex with INAD. **(B)** In the presence of light, CRY can bind INAD directly. In the presence of both light and calmodulin, CRY–INAD binding is strengthened, thus consolidating the initial light-induced response.

An important component of the Signalplex is calmodulin (CaM), which binds to INAD (Chevesich et al., 1997; Tsunoda et al., 1997; Xu et al., 1998), NINAC, TRP, and TRPL channels (Phillips et al., 1992; Warr and Kelly, 1996), and the rhodopsin phosphatase Retinal degeneration C (RdgC), inducing photoresponse termination (Lee and Montell, 2001). We have identified and characterized a functional CaM binding motif in the CRY CTT and demonstrated that CaM bridges CRY and INAD, forming a ternary complex *in vivo* (Mazzotta et al., 2018). We therefore hypothesized that the light-dependent CRY function in the photoreceptors may consist of fast and slow responses: a rapid light response, mediated by CRY conformational changes, would stimulate the direct interaction with INAD, and a novel, slower mechanism regulated by CaM would enhance its functional response (Mazzotta et al., 2018; Figure 4).

Cryptochrome interaction with the visual signaling cascade at the membrane of photoreceptor cells appears to enhance photosensitivity, especially during the night, perhaps by strengthening the interaction between INAD, NINAC, and F-actin and thus increasing the activation of TRP channels (Mazzotta et al., 2013). CRY in photoreceptor cells ultimately modulates circadian visual sensitivity: indeed, while wild-type flies show maximal sensitivity (measured by electroretinogram, ERG) in the first part of the night (Chen et al., 1992), in *cry* mutants, such sensitivity does not depend on the time of day (Mazzotta et al., 2013). Similar results are observed for optomotor turning response (Barth et al., 2010; Mazzotta et al., 2013), and rescue experiments show that this effect is specific for CRY expressed only in photoreceptors (Mazzotta et al., 2013). Moreover, flies expressing constitutively active CRY (CRYΔ) show optomotor turning response at very low levels, as a result of an impairment in either detecting movements or processing information (Damulewicz et al., 2017). Indeed, we have observed an involvement of CRY in the light-dependent degradation of the presynaptic scaffolding protein Bruchpilot (BRP), its direct partner in the photoreceptor terminals within the lamina (Damulewicz et al., 2017). The daily pattern of BRP in tetrad synapses in the distal lamina (Meinertzhagen and O’Neil, 1991; Górska-Andrzejak et al., 2013) is altered in *cry*^0^ mutants, with higher levels during the day; by contrast, in CRYΔ-overexpressing flies, the daily pattern of BRP is maintained, albeit with extremely low levels of protein (Damulewicz et al., 2017).

We have shown that in the rhabdomeres, CRY interacts also with F-actin, probably reinforcing the binding of the phototransduction cascade signaling components to the rhabdomere cytoskeleton (Schlichting et al., 2018). CRY/F-actin interaction is enhanced by light, but it exists also during darkness, keeping the Signalplex close to the membrane and ready for activation during the night (Schlichting et al., 2018). Furthermore, the strong affinity of CRY for F-actin could also prevent its degradation through the proteasome: indeed, in the rhabdomeres, CRY is not degraded by light, while in the somata of photoreceptors cells, its levels strongly decrease after light exposure (Schlichting et al., 2018). CRY in the photoreceptor cells is involved in the ability of flies to entrain their locomotor behavior to red-light cycles, a role that is largely independent of its photoreceptive function, since red light is not able to induce CRY-mediated photoresetting of the clock (Schlichting et al., 2018).

## CRY and Neuronal Activity: UV-Light Response and Arousal

*Drosophila* l-LN_v_s show higher daytime light-driven spontaneous action potential firing rate: this electrophysiological response is attenuated either in the *cry*^b^ hypomorphic mutant or in flies with disrupted opsin-based phototransduction (Sheeba et al., 2008; Fogle et al., 2015) and completely abolished in *cry*^0^ flies (Fogle et al., 2011) but is functionally rescued by targeted expression of CRY in the l-LN_v_s (Fogle et al., 2015). Indeed, these neurons undergo a CRY-dependent rapid membrane depolarization and augmented spontaneous action potential firing rate upon illumination with blue light (Fogle et al., 2011). CRY is involved in membrane depolarization by a redox-based mechanism mediated by potassium channel heteromultimeric complexes consisting of redox sensor potassium channel beta-subunit (Kvβ) HYPERKINETIC (Hk) and other channels such as Shaker, Ether-a-go-go, and Ether-a-go-go-related gene (Fogle et al., 2015; Hong et al., 2018). Interestingly, the expression of CRY in innately light-insensitive neurons renders them light responsive (Fogle et al., 2011). Furthermore, it is worth noticing that such CRY-mediated light response, involving a flavin redox-based mechanism and relying on potassium channel conductance, is independent of the circadian interaction of CRY with TIM (Fogle et al., 2011). Also, in non-neural tissues, like salivary glands, which lack a peripheral clock, CRY maintains high membrane input resistance in an Hk, Shaker, and Ether-a-go-go-dependent but light-independent manner (Agrawal et al., 2017). Very interestingly, it was recently reported that light-evoked CRY membrane electrical depolarization involves W420, located in proximity to CRY FAD and important for CRY-mediated depolarization in responses to not only UV and blue but also red light, at relatively low light intensity (Baik et al., 2019).

The electric activity of l-LN_v_s triggers two circadian behaviors in *Drosophila*: UV light avoidance/attraction and sleep/arousal (Baik et al., 2017, 2018).

Like several insects, *Drosophila* shows a rhythmic short-wavelength (UV) light avoidance, a physiological and behavioral response to sunlight which is essential for survival. This peak of UV avoidance coincides with siesta in adult flies and with peak UV light intensity in the environment (Baik et al., 2017). CRY mediates the l-LN_v_s electrophysiological response to UV light: indeed, it is significantly attenuated in *cry*^0^ and *hk*^0^ mutant flies and rescued by LN_v_-targeted expression of CRY (Baik et al., 2017, 2018).

In l-LN_v_s, CRY is also involved in the dopamine signaling pathway responsible for acute arousal upon sensory stimulation. Indeed, the clock mutant *Clk*^Jrk^ flies, which exhibit nocturnal behavior and a clock-independent reduction in total sleep time (Kim et al., 2002; Lu et al., 2008), also display high levels of CRY, which drive nighttime activity (Kumar et al., 2012). This nocturnal behavior of *Clk*^Jrk^ mutants largely depends on increased dopamine, since it is suppressed by blocking dopamine signaling, either pharmacologically or genetically (Kumar et al., 2012). High levels of dopamine act as a trigger to activate CRY, which promotes nocturnal activity. This role of CRY is limited to the night since light induces either CRY degradation (Lin et al., 2001) or the inhibition of dopamine signaling (Shang et al., 2011).

## CRY and the Regulation of Metabolic Processes

Wild-type flies show a rhythmic feeding behavior, which is under circadian and homeostatic control and depends on light exposure and food availability (Xu et al., 2008). Under LD cycles, flies show a feeding peak at ZT 0–2; this rhythm is maintained in DD, but a late-evening feeding bout is observed at CT20-4 (Seay and Thummel, 2011). Although endogenous, the rhythm is regulated by light, and CRY has been identified as the light-signaling factor involved in suppression of the evening feed activity observed in DD (Xu et al., 2008). Indeed, in LD, *cry* mutants exhibit the early morning feeding activity displayed by wild-type flies, but in addition, they also show the late-evening feeding activity, similar to that of wild-type flies in DD (Xu et al., 2008). However, this role of light-activated CRY is not dependent on light-induced TIM degradation, since *tim* mutants in LD do not show the evening feeding activity (Xu et al., 2008).

Cryptochrome function is also important for metabolic processes and carbohydrate homeostasis. Indeed, in LD-entrained wild-type flies, trehalose, the predominant circulating form of sugar in flies, is at its lowest values at the beginning of the day and increases to up 80% 4 h after feeding. Most of this sugar is confined as stored energy, and glycogen levels reach maximum values at the end of the day, accordingly (Seay and Thummel, 2011). The oscillation in glycogen concentrations is a clock-dependent process as, although dampened, it persists in constant conditions, while a clear rhythm is absent in *tim* mutants in both LD and DD (Seay and Thummel, 2011). The phase of glycogen accumulation is significantly anticipated in *cry*^01^ flies entrained in LD, indicating the involvement of light-activated CRY in setting the phase of this oscillation, and this observation is further supported by the dampened oscillation of glycogen accumulation observed in DD, when CRY is not activated by light (Seay and Thummel, 2011). This metabolic alteration observed in *cry*^01^ flies, which still possess a functioning clock, indicates that the role of CRY in setting the phase of accumulation and utilization of glycogen is not related to the canonical clock function.

In mammals, CRY1 is also involved in the regulation of gluconeogenesis by CREB/cAMP signaling through rhythmic repression of glucocorticoid receptor and decreasing the level of nuclear FoxO1 (Hatori and Panda, 2010; Zhang E.E. et al., 2010; Lamia et al., 2011; Jang et al., 2016). Moreover, CRY1 interacts with the autophagosome marker light chain 3 (LC3), responsible for its time-dependent autophagic degradation (Toledo et al., 2018). (LC3)-interacting region (LIR) motifs are found in the CRY1 sequence, and their role has been confirmed by the observation that mice in which autophagy is genetically blocked exhibit accumulation of CRY1 and disruption of the circadian clock in the liver (Toledo et al., 2018). Moreover, autophagic degradation of CRY1 is important in maintaining blood glucose levels by driving gluconeogenesis (Toledo et al., 2018). As in mammals (Turek et al., 2005; Green et al., 2008), the circadian clock is involved in fat storage and mobilization also in *Drosophila*. Indeed, a significantly reduced triacylglycerol concentration is observed in *tim*^0^ compared to wild-type LD-entrained flies (Seay and Thummel, 2011), and an altered *Clk* function in the PDF neurons results in increased fat body triglycerides (DiAngelo et al., 2011). On the other hand, a significant reduction in triacylglycerol levels is observed in both *cry* mutants reared in LD and in wild-type flies after 2 days of DD compared to LD, indicating that also light input seems to be necessary for lipid homeostasis (Seay and Thummel, 2011). In mammals, *Cry1* mutation does not significantly affect triglycerides and fatty acid blood levels (Griebel et al., 2014), while *Cry1/2*-deficient mice exhibit increased insulin secretion and lipid storage in the adipose tissue under a high-fat diet (Barclay et al., 2013).

## CRY and Aging

Aging is a process affecting most physiological processes. The circadian clock plays an important role in the aging processes: indeed, its disruption leads to accelerated aging in animals (Davidson et al., 2006; Kondratov et al., 2006; Antoch et al., 2008; Yu and Weaver, 2011), and older individuals show decreased amplitude of clock gene oscillation and changes in rhythmicity, that is, sleep/wake cycles and hormonal fluctuations (Valentinuzzi et al., 1997; Huang et al., 2002; Hofman and Swaab, 2006; Kondratova and Kondratov, 2012). Similar effects are observed in *Drosophila*, where clock mutants exhibit increased oxidative stress levels and neurodegeneration (Krishnan et al., 2009, 2012) and changes in sleep patterns and clock gene expression amplitude are observed in older flies (Koh et al., 2006a; Luo et al., 2012; Rakshit et al., 2012; Umezaki et al., 2012; Solovev et al., 2019). It has been reported that CRY is reduced at both mRNA and protein levels in the heads of older flies and that its overexpression in the nervous system or in all clock-expressing cells is able to increase the amplitude of clock gene expression levels and survival under hypoxia (Rakshit and Giebultowicz, 2013; Solovev et al., 2019). *cry*^0^ flies exhibit an accelerated functional decline, in terms of decreased climbing activity, accumulation of oxidatively damaged proteins and reduced health span (Rakshit and Giebultowicz, 2013; Solovev et al., 2019). CRY overexpression in the entire nervous system and in both central and peripheral oscillators maintains the rhythmicity of locomotor activity, increases climbing performance, and decreases recovery time after short-term hypoxia in older flies (Rakshit and Giebultowicz, 2013; Solovev et al., 2019). Nevertheless, the overexpression of CRY limited to clock neurons is not sufficient to slow down the aging processes or to reverse age-associated phenotypes (Rakshit and Giebultowicz, 2013).

## Conclusion

**FIGURE 5:**
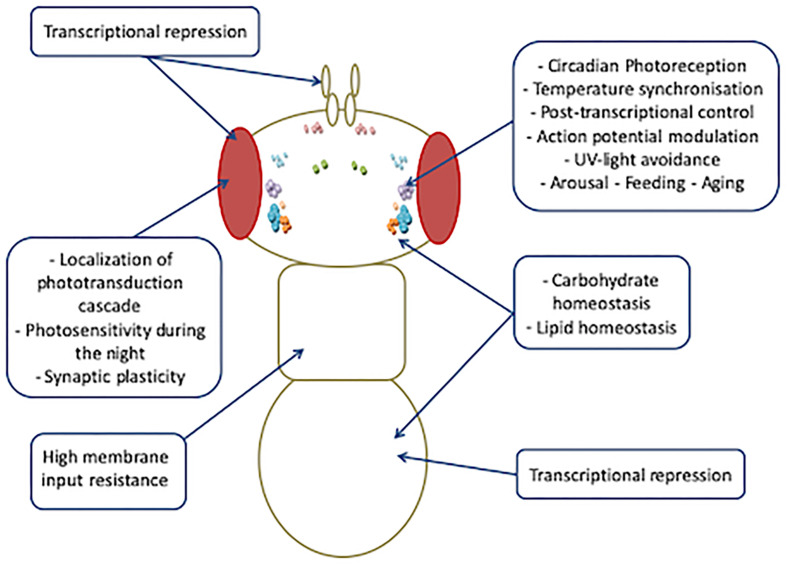
Overview of tissue-specific activities of CRY.

Increasing evidence indicates that the spectrum of biological functions of *Drosophila* CRY is wider than that exerted in circadian clocks (Figure 5).

More intriguingly, all such photoreceptor-independent roles of CRY seem to be cell or tissue specific, and different regulating mechanisms might account for the high versatility of its functioning. At least four different tissue-specific regulation mechanisms could make CRY pleiotropy possible: (1) In the clock neurons, the blue light-dependent FAD photoreduction induces conformational changes in the Trp tetrad, which results in the displacement of the CTT from the photolyase homology domain and in consequent protein activation (Zoltowski et al., 2011; Czarna et al., 2013; Levy et al., 2013; Vaidya et al., 2013; Masiero et al., 2014; Lin et al., 2018). (2) In the l-LN_v_s, light-evoked CRY membrane electrical depolarization involves W420, which is located closest to CRY FAD and is important for CRY-mediated depolarization in response not only to UV and blue light but also to red light, at a relatively low intensity (Baik et al., 2019). (3) Also in the l-LN_v_s, the CRY-mediated nocturnal activity of *Clk* mutant flies largely depends on dopamine signaling that increases CRY levels and switches these cells, which normally promote arousal in response to light, to nocturnal behavior (Kumar et al., 2012). (4) In the photoreceptor cells, CRY interacts with CaM in a Ca^2+^-dependent and light-independent manner. We have hypothesized this interaction to be functional to a Ca^2+^–CaM-dependent activation that would enhance the light-dependent CRY response (Mazzotta et al., 2018). It is possible that this mechanism might not be restricted to the photoreceptor cells, and further studies are needed to investigate whether a Ca^2+^–CaM-dependent mechanism might account for the activation/regulation of CRY activity in roles other than photoreception.

The versatility of CRY functioning in *Drosophila* shows several similarities with vertebrate CRYs that, besides being negative autoregulators of the circadian clock, also act as second messengers between the core clock and other cellular processes, such as maintenance of cellular and genomic integrity, and metabolism (Van Der Horst et al., 1999; Shearman et al., 2000; Hirayama et al., 2003; Kiyohara et al., 2006; Sato et al., 2006; McCarthy et al., 2009; Kang et al., 2010; Lamia et al., 2011; Narasimamurthy et al., 2012; Kang and Leem, 2014; Papp et al., 2015).

However, the nature of the transduction signaling involving CRYs remains largely unknown. Further studies, aimed at identifying the signal transduction underlying light-independent CRY functions, will help to improve the understanding of the biology of circadian rhythm regulation.

## Author Contributions

MD and GM wrote the manuscript.

## Conflict of Interest

The authors declare that the research was conducted in the absence of any commercial or financial relationships that could be construed as a potential conflict of interest.
