# Corrigendum: One Actor, Multiple Roles: The Performances of Cryptochrome in *Drosophila*

**DOI:** 10.3389/fphys.2020.00841

**Published:** 2020-07-28

**Authors:** Milena Damulewicz, Gabriella M. Mazzotta

**Affiliations:** ^1^Department of Cell Biology and Imaging, Jagiellonian University, Kraków, Poland; ^2^Department of Biology, University of Padua, Padua, Italy

**Keywords:** cryptochrome, *Drosophila*, circadian clock, phototransduction, circadian plasticity, light-independent activity

There is an error in the Funding statement. In the original article, we included by mistake the following funders: **CINCHRON: Comparative INsect CHRONobiology_European Union's Horizon 2020 under the Marie Skłodowska-Curie grant agreement no. 765937; National Research Council of Italy (EPIGEN Progetto Bandiera Epigenomica – Subproject 4) to GM**. These funders should be removed from the Funding Statement.

The authors apologize for these errors and state that they do not change the scientific conclusions of the article in any way. The original article has been updated.

